# Challenges and constraints to the sustainability of poultry farming in Republic of Korea

**DOI:** 10.5713/ab.24.0641

**Published:** 2025-02-25

**Authors:** Sidong Kim

**Affiliations:** 1 Poultry Research Institute, National Institute of Animal Science (NIAS), Rural Development Administration (RDA), Pyeongchang, Korea

**Keywords:** Avian Influenza, Breeding Stock, Climate Change, Farm Succession, Market Transparency, Sustainable Poultry Production

## Abstract

As of 2022, the Republic of Korea accounted for 0.8% of global chicken meat production and 0.9% of global egg production. The country achieved self-sufficiency rates of 83.1% for chicken meat and 99.4% for eggs, demonstrating significant quantitative and qualitative growth to meet domestic demand. Although the industry is trending towards expansion and specialization, it faces several challenges in achieving sustainable poultry production. Key challenges in Korea include highly pathogenic avian influenza and pest issues, climate change and the push for carbon neutrality, reliance on imported breeding stock, insufficient preparedness for expanding cage space per laying hen, post-settlement payment systems for egg sales and an oversupply of chicken meat, and the aging poultry farming population and the closure of farms unable to secure successors. Following strategies are proposed to overcome or mitigate challenges mentioned above: (1) enhancing farm biosecurity and implementing vaccination policies for disease control, (2) modernizing facilities and promoting carbon-neutral practices to adapt to climate change, (3) diversifying breeding stocks across multiple locations and developing domestic strains, (4) implementing policies and supporting farms based on a comprehensive readiness assessment of all farms regarding expanded cage space requirements, (5) improving market transparency for the egg industry and regulating supply and demand in the broiler industry, and (6) offering incentives for farm succession, attracting labor, and promoting coexistence between corporations, rural communities, and small farms. In conclusion, the sustainable development of Korea's poultry industry is not a simple task. It requires a comprehensive approach considering economic efficiency, animal welfare, environmental protection, food security, and the symbiosis with rural communities. This approach necessitates efficient cooperation among all stakeholders, including the government, farmers, integrators, retailers, and research institutions, along with a comprehensive, phased strategy for both short- and long-term goals.

## INTRODUCTION

The poultry industry in the Republic of Korea serves as a vital source of protein for the Korean consumers and significantly contributes to the national economy. Although Korea's share of global poultry meat production is relatively small [1], the industry has experienced substantial growth in both quantitative and qualitative, effectively meeting the increasing domestic demand.

From a food security standpoint, the poultry industry has made significant progress. Currently, the self-sufficiency rate for chicken meat surpasses 83%, while egg production is nearly 100% [2]. These high self-sufficiency rates highlight the industry's capacity to meet domestic needs and reliance on imports, thereby enhancing national food security.

However, the Korean poultry industry faces several challenges. These include risks from diseases such as highly pathogenic avian influenza (HPAI), extreme weather events related to climate change, reliance on imported breeding stock, rising social expectations for animal welfare, complex distribution systems, and an aging rural population. Each of these factors threatens the sustainability of the poultry industry.

This study aims to analyze the current state of Korea's poultry industry, identify its major constraints, and propose solutions to ensure its continued sustainability.

## CURRENT STATE OF THE POULTRY INDUSTRY IN THE REPUBLIC OF KOREA

### Korea's share in the global poultry industry

**Figure 1:**
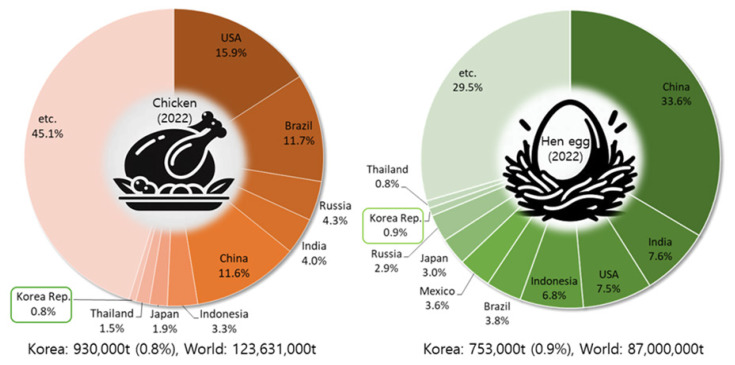
World and Korean chicken and egg production in 2022. Data from Kim et al [1].

In 2022, as shown in Figure 1, global chicken meat production was 123.6 million tons, with Korea producing 0.93 million tons, accounting for 0.8% of the total. World egg production in 2022 was 87 million tons, of which Korea produced 0.75 million tons, representing 0.9% [1]. The global chicken meat import value in 2022 was $26.49 billion. Major importing countries included China (15.5%), the UK (6.7%), France (6.1%), Germany (6.0%), Japan (5.8%), and the Netherlands (4.9%). Korea's chicken meat imports in 2022 were valued at $449.73 million, about 1.7% of the global total [1].

The self-sufficiency rate for chicken meat in Korea has been over 80% since 2005 (Table 1). In 2022, Korea produced 706,000 tons of eggs domestically and imported only 7,036 tons of fresh eggs, egg whites, and egg yolks. Table 1 also indicates the low reliance on imports for eggs, as the self-sufficiency rate ranged between 99% and 100% for the last two decades [2]. The slight decrease in self-sufficiency rate since 2020 was due to the impact of HPAI in Korea.

### The share of livestock and poultry industries in Korean agriculture

Table 2 presents the statistics for agricultural and livestock production values in Korea from 1990 to 2022 [3]. In 2022, Korea's agricultural production value was 57.9 trillion won. The total livestock production value of 25.2 trillion won represented 43.6% of the total agricultural production value. Notably, the share of livestock within agricultural production increased from 22.0% in 1990 to 43.6% in 2022. Additionally, the production shares of chicken, duck, eggs, and quail eggs have increased. In 1990, their shares were 2.5%, 0.2%, 2.3%, and 0.0%, respectively. By 2022, these shares rose to 4.5%, 1.9%, 4.5%, and 0.1%. This indicates that the poultry industry's share within the livestock sector has doubled over the past three decades, increasing from 5% in 1990.

### Changes in poultry population and farming households

**Figure 2:**
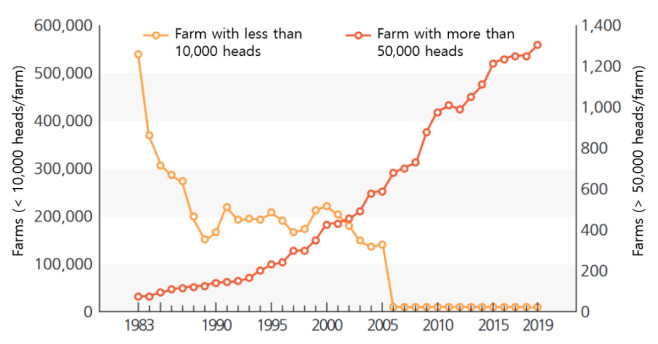
Trends of number of farms by flock size (including both broiler and layer farms). Adapted from National Geographic Information Institute [14] with CC-BY-NC-ND.

An observation of the changes in the poultry population and the number of farming households (Table 3) reveals that the numbers of laying hens, broiler chickens, and ducks have steadily increased from 42.4 million, 26.9 million, and 717 thousand in 1990 to 74.2 million, 88.7 million, and 6.0 million in 2022, respectively [4]. However, the number of farms raising these birds has dramatically decreased from 3,932, 3,547, and 13,804 in 1990 to 937, 1,454, and 338 in 2022 for laying hens, broiler chickens, and ducks, respectively. As shown in Figure 2, while the number of farms raising fewer than 10,000 birds has sharply declined, farms with over 50,000 birds have continued to increase [5]. This trend of farm scaling and specialization is expected to continue. Meanwhile, there has been a significant decrease in birds and farms for ducks since 2015. This is mainly attributed to the worsening farming conditions, including the almost annual occurrence of HPAI since 2010 and the implementation of winter season restrictions (WSR) starting in 2017. Besides chickens and ducks, other poultry species are raised on a smaller scale (Table 4) [6].

The Animal Welfare Livestock Farm Certification program has been implemented since 2012, based on the grounds established by revising the "Animal Protection Act" in 2011. As of 2023, according to the National Animal Welfare Information System [7], 240 laying hen farms have been certified, representing 6.9% of the total laying hen population of 77.20 million in 2023. For broiler chickens, 150 farms have been certified by 2023, accounting for 12.0% of the total broiler chicken population of 94.10 million in 2023.

### Changes in poultry meat and egg production and consumption

**Figure 3:**
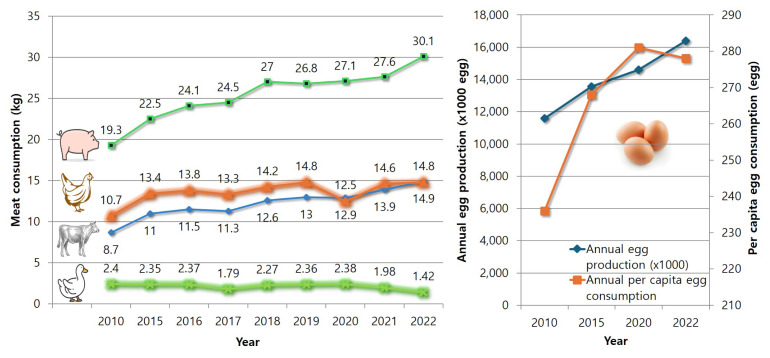
Trends of per capita meat and egg consumption. Data from Ministry of Agriculture, Food and Rural Affairs [8] and Korea Duck Association [9].

Figure 3 shows the per capita consumption of chicken and duck meat in Korea. The per capita beef, pork, and chicken consumption has increased [8]. Comparing 2022 to 2010, the per capita consumption of beef increased from 8.7to 14.9 kg (171%), pork from 19.3 to 30.1 kg (156%), and chicken from 10.7 to 14.8 kg (138%). However, duck meat consumption decreased from 2.4 kg in 2010 to 1.42 kg in 2022 (59%) [9]. This decrease is due to the continued occurrence of HPAI since 2010 and the restrictions on duck farming during winter, which have limited production. Like the increase in meat consumption, egg production and consumption have continued to rise. As shown in Figure 3, annual egg production increased from 11.6 billion eggs in 2010 to 16.4 billion eggs in 2022, a 42% increase. The annual per capita egg consumption also increased from 236 eggs in 2010 to 278 eggs in 2022, an 18% increase [8].

## MAJOR CHALLENGES AND MEASURES FOR SUSTAINABILITY OF THE POULTRY INDUSTRY

### Highly pathogenic avian influenza

**Figure 4:**
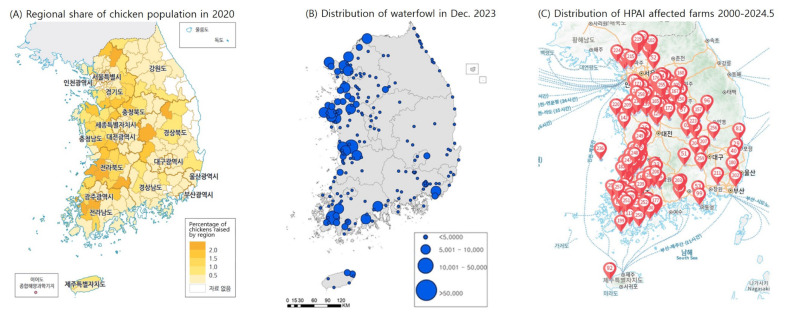
Spatial distribution of poultry population, waterfowl, and HPAI-affected farms in Korea. Adapted from National Geographic Information Institute [14] with CC-BY-NC-ND, Ministry of Environment [15] with CC-BY, and Ministry of Agriculture, Food and Rural Affairs [16] with CC-BY-NC-ND.

HPAI is the largest disease threat to the sustainable poultry industry in the world. This disease has been reported to spread between wild birds and poultry and to over 43 mammal species [10]. Recently, cases of cat infections have been reported in countries such as Korea, France, the US, and Italy [10]. Notably, in 2024, the US confirmed HPAI transmission in dairy cattle, with cross-species transmission observed between cattle, cats, poultry, and humans [11]. This underscores the need for enhanced HPAI management in the poultry industry. HPAI-infected migratory birds can introduce the virus while traveling along the Asia-Australia Flyway through Korea to Southeast Asia or when returning north [12]. When analyzing risk factors for AI infection in poultry farms, reservoirs or rivers near farms increased the cross-risk by 2.89 times, and AI outbreaks in neighboring farms increased the risk by 4.54 times. Properly disinfected farms showed a reduced risk of 0.54 times [13]. As seen in Figure 4, chicken and duck farms are concentrated in the west coast region (A) [14], which also has a high concentration of migratory waterfowl in winter (B) [15]. The locations of actual HPAI outbreaks (C) [16] show that Korean poultry farms are constantly exposed to HPAI risk in a geographical environment. Wild birds (28%) and vehicle visits by farm owners (27%) were the primary sources of infection entering farms. Within the farm, contaminated farm owners (47%) and wild bird intrusions (18%) were the primary sources of infection [17].

To minimize the economic damage caused by HPAI, relocating farms to areas with fewer migratory birds could be effective, but this requires policy support due to socio-economic burdens. Efficient disease control measures include early detection through winter migratory bird AI surveillance and the national animal health monitoring system, preventing farm-to-farm transmission, and setting up quarantine zones [18]. Multiple approaches are needed to prevent disease entry into farms, such as modernizing farm facilities, biosecurity consulting, controlling farm owner access, preventing wild bird intrusions, and proper farm disinfection. Along with biosecurity, HPAI vaccination policies should be considered. Following France's 2023 duck vaccination program [19], vaccinating poultry within a 3-km radius of outbreak farms could minimize culling, reduce economic losses and address environmental and ethical concerns. However, as vaccination may lead to HPAI becoming endemic with silent transmission, a detailed monitoring system for vaccinated populations should also be considered [20].

Since 2017, duck farms have faced restrictions on rearing during the winter season (from November to February) in areas at high risk for HPAI, as designated by the Ministry of Agriculture, Food and Rural Affairs (MAFRA). In 2023, 59.9% of all duck farms were affected by the WSR, which impacted 19.9% of the total slaughtered ducks (Table 5) [20]. As a result, it became nearly impossible for these farms to operate normally during the winter [21]. On the other hand, WSR does not provide a lasting solution to the recurring HPAI problem. A study conducted in 2019 on improving duck farming facilities found that from 2010 to 2023, there were eleven HPAI outbreaks, with ten (90.9%) first occurring in ducks. According to the study Lee [22], 76.3% of the 910 duck farms surveyed used greenhouse-style housing, and 85.7% of the farms that experienced HPAI outbreaks had greenhouse-style housing (Table 6). This indicates that upgrading duck facilities could be a better way to prevent HPAI and sustain the duck industry than continuing with WSR. Lastly, instead of putting all the responsibility for disease control on the farms, more support, such as education and consulting on biosecurity practices, should be provided to help them prevent future outbreaks.

### Pests (red mites and darkling beetles)

In 2016, the Korean Association of Poultry Veterinarians surveyed 120 laying hen farms and found that 94% of the farms were infected with chicken red mites [23]. Chicken mites cause anemia and stress in chickens, reducing egg production by up to 20%, and are also a major cause of poultry typhoid, which significantly impacts productivity [24]. Since chicken mites can survive for 8 months without chickens [25], developing and applying Integrated Pest Management (IPM) techniques is necessary rather than attempting complete eradication.

Meanwhile, lesser mealworms (darkling beetle larvae) are a significant problem in broiler chickens. These worms gnaw on insulation materials, reducing their effectiveness by 20% to 30% and causing injuries and stress to the chickens. Additionally, when chickens consume adult darkling beetles, their feed conversion ratio increases. A 2018 survey of 25 broiler farms in Korea found that all the farms were infested with A. diaperinus, with both adult beetles and larvae found on the walls and floors, and in the central areas of the housing, under heaters and waterers [26]. It has been reported that the occurrence of these beetles is related to the reuse of litter, and their control can be achieved by limiting litter reuse, heat-treating litter, and thoroughly washing and drying poultry houses [27].

In Korea, even though a system with a legal basis was established in 2018 to support farms with mite control through a program involving professional pest control companies and government-funded initiatives, the concept of IPM for managing mites has yet to be fully established [28]. Recent cases of Darkling beetle larvae found in commercially sold chickens have highlighted Darkling beetle control as a pressing issue for broiler farms [29]. However, effective control methods suitable for local farms have yet to be developed, and government-level support, similar to that provided for mite control, has not been extended to address this issue. Therefore, it is necessary to develop a standardized IPM for both red mites and Darkling beetles and to expand the scope of government support beyond current levels.

### Climate change and carbon neutrality

According to the Republic of Korea's Adaptation Communication [30], the country's average annual temperature has risen by about 1.6°C over the past 109 years (1912–2020), higher than the global average increase of 1.09°C. Since records began in 1973, the average number of heat wave days over the past 10 years has been 14.25 days, which is about 1.5 times more than the previous period's average of 9.25 days [31]. The correlation coefficient between the insurance loss amount for poultry and the number of heat wave days is 98.6%, indicating that poultry is particularly vulnerable to heat waves [32].

To address climate change, in the short term, expanding livestock disaster insurance coverage and encouraging more farms to enroll can help farmers recover from losses caused by extreme weather events. In the long term, reducing vulnerability to climate change requires modernizing poultry facilities through climate-control systems, implementing smart farm technologies, and developing adaptive feeding and management practices. Additionally, research and policy support focused on early warning systems and preventive technologies are essential to addressing extreme weather events like heat waves [33].

MAFRA has established the 'Livestock Sector 2030 Greenhouse Gas (GHG) Reduction and Green Growth Strategy' [34]. As of 2020, GHG emissions from the livestock sector were 9.7 million tons (CO_2_-eq), which is 1.5% of the country's total emissions (656 million tons) but accounts for 46.2% of the agricultural sector's emissions (21.1 million tons). MAFRA's 2030 strategic goal is to reduce GHG emissions to 7.73 million tons, an 18% decrease from 9.4 million tons in 2018 (Table 7).

According to MAFRA's GHG reduction strategy [34], the livestock sector is divided into two stages. First, the 'livestock management stage' involves improving livestock feeding management. Second, the 'livestock manure treatment stage' focuses on improving manure treatment methods. Table 8 shows the annual GHG emissions by livestock species generated during the manure treatment process as of 2020. The poultry sector has lower GHG emissions compared to other livestock, but methane accounts for 22.4% and nitrous oxide for 14.3%. Therefore, it is necessary to broaden and propagate manure composting methods that produce less methane and provide appropriate protein feed to reduce nitrous oxide emissions. Additionally, to support carbon neutrality, the plan includes transforming the livestock industry into a low-carbon structure by expanding smart livestock farming and recycling by-products. To encourage active participation, the government plans to promote a low-carbon livestock product certification system and establish an emissions trading platform.

To achieve the carbon neutrality goal, several steps are necessary [35]: (1) developing GHG reduction technologies like effective low-methane and low-protein feeds, (2) offering incentives to encourage active participation of livestock farms and promoting 'low-carbon certified livestock products,' (3) ensuring that reduction measures are practical for farms, and (4) establishing and implementing policies that can reduce GHG while also supporting the continued development of the livestock industry.

### Dependence on imported breeding stock

The global poultry breeding industry is dominated by three multinational companies [36]. For broilers, Erich Wesjohann Group (EW Group) and Tyson Foods are the leading suppliers, while for layers, EW Group and Hendrix Genetics provide most of the world's breeding stock (Table 9). Korean farms rely almost entirely on imported breeding stock for both broilers and layers. As shown in Table 10, of the approximately 7 million breeding chickens stocked in 2021, 96.1% were imported breeds, with Arbor Acres accounting for 53.1%, followed by Ross at 30.0% and Indian River at 13.0%. The remaining 3.9% were native Korean chickens. For layers, 100% were imported breeds, with Hy-Line, ISA Brown, Lohmann Brown, Lohmann White, and Tetra Brown, of which Hy-Line accounted for 49.2% [37].

Excluding native chickens, four companies import broiler grandparent stock (GPS), one imports layer GPS, and one imports duck GPS in Korea. If any of the above companies were to face culling due to diseases like HPAI, it could lose 10% to 40% of domestic broiler GPS, 100% of layer GPS, and 100% of duck GPS. A particular concern is that even with substantial investments in disease prevention, these breeding companies remain vulnerable to preventive culling if HPAI occurs in nearby farms. The HPAI problem extends beyond domestic outbreaks. In 2014, HPAI outbreaks in the US and UK led to import bans on broiler GPS, forcing a shift to French imports and disrupting the breeding stock supply. Similarly, in 2021, duck breeding stock imports were banned due to HPAI in the UK, causing significant industry challenges. If import suspensions last over a year, the effects could potentially lead to the collapse of the domestic chicken and duck industries within 2 to 3 years. For the sustainability of the domestic poultry industry, ensuring a stable supply of breeding stock is crucial.

Therefore, efforts and support are needed at the private, academic, and government levels to address the following issues:

(1) Dispersed GPS flocks: To reduce the impact of HPAI outbreaks, it is necessary to geographically separate GPS flocks of different ages, even within a single company. However, this approach is inefficient for managing under normal conditions, so institutional and financial support from the government is essential.
(2) Careful preventive culling of GPS farms: Preventive culling by local governments in response to disease outbreaks on nearby farms should consider the biosecurity level, geographical location, and environment of GPS farms. To support this approach, a central government legal framework should be established to facilitate culling decisions that minimize the impact of GPS flock losses.
(3) Developing domestic breeding stocks: Active investment in breeding native chicken breeds can improve their production performance and reduce the dependency on imported breeding stocks.

These efforts will contribute to a more resilient and sustainable poultry industry in Korea.

### Lack of preparedness for expansion of laying hen cage space

The minimum cage space per laying hen was first implemented in 2002 with the introduction of the livestock farming registration system, setting the standard at 0.042 m^2^ per hen for registered farms. In 2013, as the registration system changed to a permit system, the space was expanded to 0.05 m^2^, effective from 2015. In 2017, a pesticide residue survey on eggs from 1,239 domestic farms found 52 farms non-compliant, raising public concern about overcrowding. Consequently, in 2018, the space per hen was increased to 0.075 m^2^. This new standard was applied to new farms from September 2018 and will be enforced for existing farms from September 2025. Additionally, in 2018, testing for residual substances in eggs and Hazard Analysis and Critical Control Point (HACCP) standards for laying hen farms became mandatory. From 2019, egg-laying date and farming environment information were required to be printed on eggshells, and in 2020, a traceability system for eggs, chicken, and duck meat was introduced, providing a secure and transparent supply chain for all stakeholders.

With the full implementation of the 0.075 m^2^ per hen standard from September 2025, a research survey was conducted by Korea Rural Economic Institute in 2023 to assess farm readiness [38]. The data were collected from 410 operational farms out of 583 farms surveyed. Among them, vertical (H-type) cages (0.05 m^2^/hen) were most common, used by 286 farms (69.8%), followed by A-type cages (0.05 m^2^/hen) on 73 farms (17.8%), and floor systems on 35 farms (8.5%) (Table 11). Only 27 farms (6.6%) used enriched cages (0.075 m^2^/hen). Although this study did not include all farms, it was somewhat representative of the total hen population, as it examined 410 out of 944 farms in Korea in 2023. This sample covered approximately 43% of all farms and represented 50.3% of the entire hen population in Korea. Only 6.6% of farms, accounting for 4.3% of hens, meet the 0.075 m^2^ space requirement, suggesting many farms will likely continue operating with existing facilities in 2025. The report analyzed three scenarios for reducing the laying hen population when new regulations are implemented in 2025. According to livestock farming permit and registration data, the laying hen population decreased by 14%. However, based on farm survey estimates, the laying hen population reduction rate was 19.4%. Based on a rough estimate considering the current cage area, the reduction rate was projected to be 33.3%. The research projected that these reduction rate scenarios would increase farm-gate egg prices by 24%, 33%, and 57%, respectively [38]. These substantial price increases will likely be challenging for consumers to handle. The challenge lies in the uncertainty about farmers' intentions to modify facilities, reduce hen numbers, or cease operations by 2025. Consequently, there is no clear plan for addressing the potential hen population decrease and price increases after September 2025. Therefore, to minimize disruption around 2025 and ensure a sustainable laying hen industry, the following measures are necessary:

(1) Assess farmers' preparedness plans: Conducting a comprehensive survey across all farms is important to evaluate current cage conditions, planned facility changes, potential hen reductions, and possible farm closures. Based on the survey results, appropriate government measures, such as providing a grace period for cages installed before 2018 until the end of their lifespan, should be implemented.
(2) Support for facility upgrades: Establishing a central government task force is also crucial in resolving potential conflicts with local communities and licensing issues during facility upgrades. Furthermore, considering the current facilities' lifespan and scale, providing low-interest loans or partial subsidies for facility changes should be considered.
(3) Public outreach to mitigate resistance to egg price increases: It is also crucial to explain that price increases during the transition period are necessary and in this transition period, all stakeholders (i.e., farmers and consumers) should share the hen welfare improvement cost.
(4) Ensure government policy reliability: Upholding the government's commitment is required to enhance animal welfare and allow appropriate egg price increases to help farmers recover their investment in facility upgrades.

### Delayed payment for egg sales

**Figure 5:**
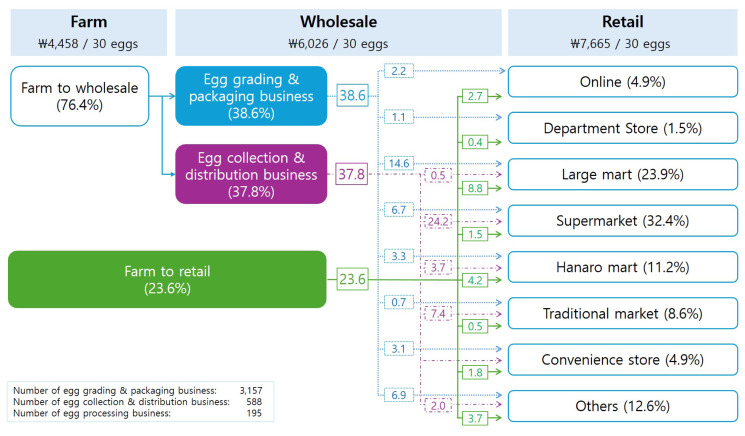
Egg distribution channels and their percentages by stage. Data from Korea Agricultural Marketing Information Service [39].

Figure 5 shows that 23.6% of eggs in Korea are shipped directly from farms to retail, mainly by large-scale farms, while 76.4% of eggs come from small-scale farms that rely on intermediate distributors [39]. These small-scale farms, which often cannot store eggs for extended periods after laying, face a disadvantage in price negotiations due to their reliance on intermediaries. Small-scale livestock farms, generally less stable and producing lower quantities, often struggle with direct transactions, making intermediary distributors essential. As of 2022, there were 3,157 edible egg grading and packaging companies and 588 edible egg collection and sales companies. These companies, as intermediaries, operate on a small and dispersed scale, limiting their market power. As a result, they often cannot pay immediately after purchasing eggs, leading to post-settlement practices becoming the norm. This situation forces farmers to supply eggs without knowing the price at delivery, making normal business operations difficult.

The absence of an egg pricing system that is mutually acceptable to farmers and distributors is the main reason for the persistence of the current post-settlement system [40]. MAFRA is attempting to establish representative pricing by supporting the creation of egg auction markets, but thus far, only three markets have been set up. Additionally, due to the regional concentration of these markets and the unique challenges associated with auctioning eggs, many farmers are hesitant to participate, primarily because they must pay listing fees. Distributors are also reluctant to engage because they must make immediate payments when purchasing eggs. These factors contribute to difficulties in establishing transparent pricing based on public auctions [40].

The agricultural and livestock market is particularly prone to information asymmetry due to the distribution chain's complexity and supermarkets' dominance in price setting, which distorts market prices [41,42]. The problem with the distribution of agricultural and livestock products is not the inefficient structure of wholesale markets or the exploitation by intermediaries but the malfunctioning of the functions that should be performed at each stage of the distribution process. When examining the margins at different stages of agricultural product distribution, it was pointed out that large retail stores at the retail stage have higher margins than at the wholesale stage, indicating a need for improving distribution efficiencies more at the retail stage than at the wholesale stage [40]. Table 12 also reveals the difference between the wholesale (14.6%) and retail margins (21.4%) for eggs, indicating a significant discrepancy in margins in the distribution of eggs [39,43,44]. Therefore, to resolve the post-settlement egg price problem, it would be more effective to involve not only intermediaries but also entities with pricing power, such as supermarkets, in efforts to improve the distribution system.

### Chicken oversupply

With the introduction of foreign broiler breeds in the 1980s, the supply of broilers increased rapidly. The typical 5-week rearing period in Korea creates sharp supply fluctuations within one to two months, leading to significant price volatility. Consequently, these price swings have resulted in economic losses for farmers. In response, the need for a livestock integration system based on contract farming emerged to assist broiler farmers raise chickens more stably, leading to the rise of integrator companies. The integration system established after 2000 involves integrator companies supplying farmers with chicks, feed, and other necessary materials while farmers raise the chicks and deliver them to the integrator companies. In return, the integrators pay farmers a fixed fee for rearing, ensuring a stable income for farmers regardless of price fluctuations while providing integrators and slaughterhouses with a stable supply of broilers [45]. As of 2021, seventy-one (71) vertically integrated broiler companies in Korea account for 94% of the total number of broilers processed [46]. The top six companies accounted for 60.9% of the total slaughter volume in the first half of 2023 [45]. Although farm integration was established, the oversupply problem persisted and became chronic. The structure of the broiler industry in Korea is a "weak oligopoly market" dominated by more than five major companies that engage in fierce competition [47]. This situation creates a "Supply Glut Dilemma," where one company's attempts to address the oversupply problem via reducing overall chick production or increasing prices is eventually caught as an opportunity for other companies to capture that market share instead of cooperating, making it difficult to resolve the problem. Consequently, the pressure to improve productivity and management efficiency has been exerted on all participants in the integration system, which has become a source of conflict between integrator companies and contract farmers.

MAFRA requested that the industry autonomously regulate supply and demand to address the above issue. However, due to the nature of the integration system, where integrators are deeply involved in production, there have been instances where the Fair Trade Commission (FTC) imposed fines on related companies and associations for collusion in 2006 and 2022. To resolve these issues, the Act on Livestock Farm Alliance Systems (2012) was enacted, allowing for the adjustment of production in consultation with the FTC, and the Livestock Industry Act (2021) established a framework for operating the Supply and Demand Adjustment Committee (SDAC). However, it may be practically challenging to regulate supply and demand quickly through consultations with the FTC. Additionally, companies that have previously been fined or investigated by the FTC may be reluctant to participate in the adjustments through the SDAC. Therefore, when operating the SDAC, it is essential to restore mutual trust among stakeholders and ensure institutional and procedural legitimacy to avoid misunderstandings of collusion. Meanwhile, the Livestock Checkoff Fund Act allows checkoff funds to stabilize supply and demand, improve distribution structures, and promote exports. Therefore, it is necessary to solidify the collection and operation of the chicken checkoff fund and use it to stabilize supply and demand through the SDAC.

On the other hand, conflicts between integrators and participating farmers have been addressed by amending the Livestock Farm Alliance Systems Act to provide the basis for standard contracts, establish farmer councils, and create dispute resolution committees. However, there are still ongoing complaints regarding issues such as breeding fees, feed quality, chick quality, contract fairness, and problems arising from the bankruptcy of integrators.

Therefore, the following efforts are needed [48,49]:

(1) Upgrading breeder farms: To enhance productivity and improve chick quality, breeder farms should transition to a direct management system by integrators, enabling better support for facility modernization and chick placement scheduling for broiler farms.
(2) Introducing quality assurance for feed and chicks: To ensure the quality of feed and chicks provided to farmers, standard contracts should include detailed feed and chick quality information.
(3) Creating a protection system for farmers in case of integrator bankruptcy: To reduce risks for farmers, a system similar to the statutory trust under the United States Packers and Stockyards Act should be established to ensure that payments to farmers are prioritized from the proceeds of broilers they raise under contract.
(4) Introducing a weight-based pricing system: The current 'Ho' system, which uses a ±50 g weight bracket, has been causing issues, particularly during HPAI-related standstill periods. Farmers may incur extra feed costs for overweight birds without compensation, and consumers may not receive consistent weights within the same 'Ho' bracket. Therefore, adjusting the 'Ho' system to a pricing model based directly on weight would be beneficial.

### Aging in rural areas and related issues

Due to aging operators and succession issues, Korea's livestock farming sector faces significant challenges. From 2005 to 2019, the proportion of farm operators aged 65 and older increased substantially. Smaller farms, particularly those with fewer than 50,000 birds, are more likely to lack successors than larger farms (Table 13). Even among larger farms, however, nearly half report having no successor [50], with various factors further complicating the succession process. These include the need for substantial scale to maintain profitability, facility renovation or expansion challenges, regulatory hurdles, and disagreements with nearby residents. High entry costs, such as land, facilities, and livestock purchases, also create barriers for new entrants. Consequently, the closure of farms without successors will inevitably reduce the number of farms and birds, potentially leading to supply issues. To address the challenges posed by the declining number of farms, it is essential to consider the unique characteristics of Korea's livestock industry [51]. Korea faces limitations in sustaining its production base through farm consolidation and centralization due to the continued decrease in farm numbers. While some countries, such as those in South America, have tackled these challenges through corporate-led production, concerns in Korea about the weakening of small farms and the potential increase in imported livestock products for corporate profit led to a legal ban on large corporate participation. Although this restriction was lifted after 2010, opposition from farms and related groups remains strong [52]. Therefore, maintaining the production base through farm succession is of utmost importance. To maintain agricultural competitiveness, it is crucial to ensure the smooth succession of farms to the next generation without reducing their scale. This includes addressing issues such as gift taxes and inheritance divisions that can lead to farm size reduction. Overall, the industry must find ways to facilitate succession within families and entry for new farmers to sustain its future.

MAFRA's Successor Farmer Selection and Support Program, initiated in 1981, aims to address succession issues in livestock farming. The program offers low-interest loans of up to 500 million won for farm development, with favorable terms including a 1.5% interest rate, 5-year grace period, and 20-year repayment schedule. While comprehensive in its support (financial aid, education, and consulting), the program has strict eligibility criteria, targeting individuals aged 18 to 50 with limited farming experience and relevant agricultural education. These conditions make the program insufficient as an effective policy measure to promote farm succession. Therefore, it is crucial that additional policy measures be implemented to effectively promote farm succession [53]:

(1) Increase farm inheritance tax deduction limit: The current tax deduction limit should be raised to facilitate the smooth succession of larger farms and agricultural corporations.
(2) Expand special tax deduction limit for agricultural gifts: To prevent excessive gift taxes during the transfer of agricultural property, it is necessary to increase the special tax deduction limit for agricultural gifts, thereby encouraging farm succession during the owner's lifetime.
(3) Promote agricultural corporatization: To address the issue of property division during inheritance, it is important to promote the corporatization of farms by converting family farms into family corporations, with family members as shareholders. This would enable farm succession through the transfer of shares, allowing for the separation of ownership and management and facilitating third-party farm succession. Importantly, to encourage third-party farm succession, institutional support is crucial, such as tax exemptions for young startup farmers who receive gifts from non-farming parents to invest in agricultural operations, including setting up livestock facilities or purchasing livestock.

Securing labor is also crucial for farm management. To mitigate the shortage of rural labor due to the aging population, it is necessary to attract young people and foreign workers and strengthen their capabilities. To attract young people, it is essential to reduce entry barriers through the related support mentioned above, provide agricultural technology education, and improve living conditions. Additionally, to ensure a stable supply of labor, the employment of foreign workers in the agricultural sector should be expanded, with support for their settlement to encourage long-term employment and integration into the local community. Furthermore, institutional improvements are needed to solve the mismatch in the supply and demand of agricultural labor in different regions and establish labor-saving facilities such as smart farms [54].

From 1968 to 1990, the government, responding to a shortage of livestock products, requested large corporations to enter the livestock industry, allowing them to own farmland and farms directly. During this period, large corporations played a crucial role in cultivating livestock experts, accumulating breeding technology and know-how, and providing opportunities for the growth of full-time farmers. However, due to conflicts with full-time farmers and issues such as real estate speculation by large corporations, the Livestock Industry Act of 1989 was enacted to restrict corporate participation in the industry. From 1990 until the lifting of related regulations in 2010, corporate participation in livestock farming was limited to vertical integration projects, and the industry was mainly led by small and medium-sized enterprises. Around 2005, as farms' financial situation deteriorated, a new form of corporate participation emerged, triggered by feed companies acquiring farms while recovering feed costs. This led to a model where feed companies began operating directly managed farms. As the number of farms decreased and the scale of farming grew, feed companies increasingly began to operate farms to survive. With the anticipated closure of farms due to a lack of successor farmers, corporate participation in livestock farming is expected to continue increasing [55].

There are concerns that corporate involvement in livestock production could accelerate the exit of family-run and small-scale farms, and if efficiency is pursued solely based on corporate logic, the traditional rural community where multiple families live and produce livestock might disappear, potentially undermining the "multifunctional role" of agriculture [55]. However, simply preventing corporations from directly participating in livestock production and focusing solely on nurturing and supporting successor farmers may not be sufficient to ensure national food security and the sustainability of the livestock industry. Therefore, it is necessary to devise strategies that promote the coexistence of corporations, rural communities, and livestock farmers. In particular, when large corporations enter the livestock sector, it is essential to establish relevant systems and support measures that encourage adopting practices such as enhanced animal welfare, Environmental, Social, and Governance management, and moving away from "factory farming." Additionally, efforts should be made to preserve and improve rural landscapes and environments and to promote coexistence and development with rural residents [56,57].

## CONCLUSION

Since 2013, Korea has implemented a livestock permit system to ensure trained owners operate farms in adequately equipped facilities. The government has also worked to enhance animal welfare by expanding cage space for layer hens in 2018. It has strengthened disease monitoring and hygiene management with a traceability system for chickens, ducks, and eggs introduced in 2020. Additionally, economic support has been provided for red mite control and smart farm infrastructure. However, the sustainability of the poultry industry cannot be achieved through regulations and financial support alone; a strategic approach is needed to address the various constraints mentioned above. The future of the Korean poultry industry must involve a comprehensive approach that considers not only economic and efficiency factors but also animal welfare, environmental protection, food safety, and coexistence with rural communities. With the cooperation of government, industry, and farmers, supported by policy measures and technological innovation, the poultry industry can maintain productivity and contribute to national development as a sustainable and essential sector. In the short term, coordinated efforts are needed to address issues such as oversupply in broiler production and delayed payments for egg sales. Broiler integrators, the government, and poultry producers using checkoff funds should collaborate to establish a fair, voluntary supply adjustment system that complies with Fair Trade regulations. Similarly, farms, intermediaries, and retailers should work with government bodies to implement timely payment practices throughout the egg supply chain, o promoting a healthy distribution system. In the long term, fostering successor farmers, managing corporate involvement in livestock production, improving animal welfare, and working towards carbon neutrality will require legal revisions and broad social consensus, which will involve the cooperation of farms, interested corporations, government agencies, local communities, and consumers to build a shared vision for sustainable development. Achieving carbon neutrality will demand investments in energy-efficient technologies, renewable energy sources, and sustainable waste management practices. Additionally, improving disease prevention, breeding stock development, and biosecurity will require collaborative investments from farms, biosecurity and breeding companies, integrators, research institutions, and the government to enhance facilities, establish effective biosecurity protocols, and advance breeding research. By distributing responsibilities across these short- and long-term goals, the Korean poultry industry can achieve a balanced approach to growth and sustainability, ensuring a stable future for both producers and consumers.

## Figures and Tables

**Table 1:** Trends of self-sufficiency rate for chicken and egg^1)^

Year	2000	2005	2010	2015	2020	2022
Chicken (%)	79.9	84.3	83.4	86.6	88.2	83.1
Egg (%)	100.0	100.0	99.7	99.7	99.4	99.4

1) Data from Korea Rural Economic Institute [2].

**Table 2:** Agricultural and livestock production output^1)^

Item	Year									

	1990		2000		2010		2020		2022	
Agricultural production	17,860	(100)	31,968	(100)	45,724	(100)	50,173	(100)	57,905	(100)
Animal production	3,923	(22.0)	8,082	(25.3)	17,471	(38.2)	20,347	(40.6)	25,224	(43.6)
Livestock and meat	2,799	(15.7)	5,864	(18.3)	13,752	(30.^1)^	16,269	(32.4)	19,889	(34.3)
Hanwoo cattle	922	(5.2)	1,879	(5.9)	4,582	(10.0)	5,725	(11.4)	6,029	(10.4)
Dairy cattle	136	(0.8)	72	(0.2)	24	(0.^1)^	79	(0.2)	58	(0.^1)^
Pork	1,174	(6.6)	2,372	(7.4)	5,323	(11.6)	7,178	(14.3)	9,650	(16.7)
Chicken	446	(2.5)	821	(2.6)	2,146	(4.7)	2,027	(4.0)	2,581	(4.5)
Duck	38	(0.2)	474	(1.5)	1,306	(2.9)	813	(1.6)	1,076	(1.9)
Goat & etc.	82	(0.5)	246	(0.8)	369	(0.8)	448	(0.9)	496	(0.9)
Livestock products	1,124	(6.3)	2,219	(6.9)	3,719	(8.^1)^	4,078	(8.^1)^	5,335	(9.2)
Egg	408	(2.3)	651	(2.0)	1,341	(2.9)	1,634	(3.3)	2,594	(4.5)
Quail egg	8	(0.0)	18	(0.^1)^	58	(0.^1)^	60	(0.^1)^	85	(0.^1)^
Milk	638	(3.6)	1,352	(4.2)	1,693	(3.7)	2,196	(4.4)	2,139	(3.7)
Honey & etc.	70	(0.4)	198	(0.6)	627	(1.4)	189	(0.4)	518	(0.9)

Values are presented as billion Korean Won (%).

1) Data from Korea Statistical Information Service [3].

**Table 3:** Trends of poultry population and number of farms by year^1)^

Variables		Year					

		1990	2000	2010	2020	2022	2023
Number of animals (Thousand head) (A)	Layer	42,430	51,076	61,691	72,580	74,188	77,202
	Broiler	26,935	45,000	77,871	94,835	88,713	94,115
	Duck	717	5,134	14,397	7,929	5,994	6,538
Farms (B)	Layer	3,932	2,601	1,535	936	937	944
	Broiler	3,547	2,013	1,763	1,597	1,454	1,546
	Duck	13,804	12,986	5,126	449	338	370
Animals per farm (Head) (A/B)	Layer	10,791	19,637	40,190	77,543	79,176	81,782
	Broiler	7,594	22,355	44,170	59,383	61,013	60,876
	Duck	52	395	2,809	17,659	17,734	17,671

Values are presented as head, house hold.

1) Data based on the fourth quarter livestock statistics survey of each year [4].

**Table 4:** Number of animals and farms for the minor poultry species in 2022^1)^

Species	Quail	Pheasant	Goose	Turkey	Ostrich
Number of animals	15,216,066	136,627	4,307	1,595	1,106
Farms	96	170	864	409	77

Values are presented as head and household, respectively.

1) Data from Ministry of Agriculture, Forestry, Rural Affairs [6].

**Table 5:** Number of farms and ducks subject to winter season rearing restrictions since 2017

Year	Farm^1)^	Annual number of slaughtered ducks^1)^	Winter season rearing restriction^2)^			

			Number of farms		Number of ducks	
		
	(A)	(B)	(C)	(C/A, %)	(D)	(D/B, %)
2017	508	46,101	260	51.2	7,950	17.2
2020	566	66,970	256	45.2	8,080	12.1
2023	519	52,811	311	59.9	10,520	19.9

1) Duck farming survey, 3rd quarter of each year [21].

2) Data from Lee [22].

**Table 6:** Survey results on the duck housing type in 2019^1)^

Housing type	Greenhouse	Truss	Sandwich panel	Others	Total
Number of farms	695	80	128	7	910
Proportion (%)	76.4	8.8	14.1	0.8	100.0

1) Data from Korea Duck Board [23].

**Table 7:** 2030 GHG reduction target by livestock species based on 2018 baseline^1)^

	GHG emission in 2018			GHG emission in 2030			Reduction	
			
Items	Total (A)	Enteric fermentation	Manure management	Total (B)	Enteric fermentation	Manure management	A − B (C)	C/A (%)
Beef cattle	4,920	3,040	1,880	4,180	3,030	1,150	740	15
Dairy cattle	1,650	1,010	640	1,260	860	400	390	24
Swine	1,760	360	1,400	1,350	370	980	410	23
Poultry	830	0	830	800	0	800	30	4
Other	250	60	190	140	40	100	110	44
Total	9,410 (100.0%)	4,470 (47.5%)	4,940 (52.5%)	7,730 (100.0%)	4,300 (55.6%)	3,430 (44.4%)	1,680	18

Values are presented as 1,000 ton CO_2_-eq (%).

1) Livestock sector 2030 greenhouse gas reduction and green growth strategy’ for carbon neutrality by 2050 [34].

**Table 8:** Annual emissions of methane and nitrous oxide from manure management practices of major livestock species for 2020^1)^

Species	Total	Cattle	Swine	Poultry^2)^
Methane (CH_4_) (Percentage)	1,339	379	705	313
	100	27.1	50.4	22.4
Nitrous oxide (N_2_O) (Percentage)	3,592	2,296	551	513
	100	63.9	15.3	14.3

Values are presented as 1,000 ton CO_2_-eq (%).

1) Data from Lee et al [35].

2) Includes layer, broiler, and duck.

**Table 9:** Leading broiler and layer genetics companies^1)^

Purpose	Company	Headquarter	Commercial strains
Broiler	Erich Wesjohann Group	Germany	Aviagen Broiler (Arbor acres, Ross, IR), Hubbard
	Tyson Food	USA	Cobb-Vantress (Cobb)
Layer	Erich Wesjohann Group	Germany	Lohmann breeders (White, Brown), Hy-line genetics, H&N international (White, Brown, Tinted), Novogen (White, Tined, Brown),
	Hendrix Genetics	Netherlands	Dekalb, Bovans, Hisex, ISA, Shaver, Babcock

1) Data based on FOODBARONS 2022 [36] and relevant company internet webpages.

**Table 10:** Number of parent stock chicks stocked in 2021^1)^

Brands (Strains)	Number of stocked chicks	Proportion (%)
Broiler parent stocks	7,293,210	100
Ross	2,185,500	30.0
Arbor acres	3,876,300	53.1
Indian river	950,700	13.0
Tojongdak^2)^	280,710	3.9
Layer parent stocks	757,579	100
Hy-line brown	372,900	49.2
ISA brown	191,360	25.3
Lohmann brown	105,959	14.0
Lohmann white	17,680	2.3
Tetra brown	69,680	9.2

1) Data based on the GPS and PS official inspection results [37].

2) Korean native or locally adapted breeds.

GPS, grandparent stock; PS, parent stock.

**Table 11:** Distribution of housing systems and flock sizes in surveyed layer farms^1)^

Housing systems	Battery cage systems			Alternative systems		
	
	H type (0.05 m^2^/hen)	A type (0.05 m^2^/hen)	Enrichable (0.075 m^2^/hen)	Aviary (≤7 hens/m^2^)	Floor (≤9 hens/m^2^)	Free range (≤9 hens/m^2^)
Farms^2^ (A)	286	73	27	9	35	13
Percentage (A/410)^2)^	69.8	17.8	6.6	2.2	8.5	3.2
Hens raised (thousand head) (B)	30,890.1	2,510.2	3,322.1	1,089.9	967.6	83.5
Percentage	79.5	6.5	8.5	2.8	2.5	0.2
National share^3)^ (%)	40.0	3.3	4.3	1.4	1.3	0.1
Hens per farm (thousand head) (A/B)	108.0	34.4	123.0	121.1	27.6	6.4

1) Data based on the survey report [38].

2) The survey allowed multiple responses. Out of a total 410 farms, 27 farms reported two types of housing systems, and 3 farms reported three types of housing systems.

3) Share of total laying hen population in 2023.

**Table 12:** Chicken and egg distribution stage prices and distribution cost rates

Items	Chicken (2022)^1)^ (Won/head)	Egg (2022)^2)^ (Won/30 eggs)	Duck (2021)^3)^ (Won/head)
Producer price (A)	2,640	4,458	9,990
Consumer price (B)	6,393	7,665	1,5375
Distribution cost (C = B−A)	3,753	3,207	5,835
Producer receipt rate (A/B, %)	41.3	58.2	65.0
Distribution cost rate (C/B, %)	58.7	41.8	35.0
Shipping stage	0	5.8	0
Wholesale stage	34.3	14.6	8.2
Retail stage	24.4	21.4	26.8

1) Domestic distribution status of chicken [43].

2) Domestic distribution status of egg (60 to 67 g/egg) [39].

3) Distribution information survey [44].

**Table 13:** Heir status of layer farms and broiler farms by farm size: results from 2014 farm survey^1)^

Species	Heir status	Farm size (heads)					

		< 30,000		30,000 to 50,000		>50,000	
		
		Farms	Percentage	Farms	Percentage	Farms	Percentage
Layer	Present	6	28.6	4	23.5	31	54.4
	Absent	15	71.4	13	76.5	26	45.6
	Total	21	100.0	17	100.0	57	100.0
Broiler	Present	2	12.5	7	25.9	32	47.8
	Absent	14	87.5	20	74.1	35	52.2
	Total	16	100.0	27	100.0	67	100.0

1) Data based on the farm survey results [50].
